# A novel plant E3 ligase stabilizes *Escherichia coli* heat shock factor σ^32^

**DOI:** 10.1038/s41598-017-03056-6

**Published:** 2017-06-22

**Authors:** Yulong Niu, Xibing Xu, Chengcheng Liu, Tao Wang, Ke Liang, Jianmei Wang, Zhibin Liu, Xufeng Li, Yi Yang

**Affiliations:** 1 Key Laboratory of Bio-resources and Eco-environment of Ministry of Education, College of Life Sciences, Sichuan University, Chengdu, P.R. China; 2 Department of Periodontics, West China Hospital of Stomatology, Sichuan University, Chengdu, P.R. China

## Abstract

The heat shock response is crucial for organisms against heat-damaged proteins and maintaining homeostasis at a high temperature. Heterologous expression of eukaryotic molecular chaperones protects *Escherichia coli* from heat stress. Here we report that expression of the plant E3 ligase BnTR1 significantly increases the thermotolerance of *E*. *coli*. Different from eukaryotic chaperones, BnTR1 expression induces the accumulation of heat shock factor σ^32^ and heat shock proteins. The active site of BnTR1 in *E*. *coli* is the zinc fingers of the RING domain, which interacts with DnaK resulting in stabilizing σ^32^. Our findings indicate the expression of BnTR1 confers thermoprotective effects on *E*. *coli* cells, and it may provide useful clues to engineer thermophilic bacterial strains.

## Introduction

The heat shock response (HSR) is a universal signalling pathway in all organisms that maintains protein-folding homeostasis through the regulation of heat shock proteins (HSPs)^1, 2^. Although the HSR varies among species, a striking common feature is the rapid induction of evolutionarily conserved HSPs, including the chaperones and proteases that perform protein refolding and degradation, thereby protecting cells from stress-induced protein misfolding or aggregation^3, 4^. In *Escherichia coli*, the HSR is a complex circuit controlled by the alternative sigma factor (σ^32^), encoded by *rpoH*, which guides RNA polymerase to HSP gene promoters in heat stress^5–7^. In the canonical *E*. *coli* HSR, HSP synthesis rapidly increases owing to the transient accumulation of σ^32^ (induction phase) and then gradually decreases during the adaptation phase to achieve a new steady state^8, 9^. During the induction phase, σ^32^ synthesis is primarily regulated at the translational level, as heat opens an inhibitory region of *rpoH* mRNA^10–12^, and σ^32^ activity and stability increase^13^. During the adaptation phase, the cytoplasmic chaperone teams DnaK/DnaJ/GrpE (KJE) and GroEL/GroES negatively regulate σ^32^ activity by sequestering σ^32^ from RNA polymerase^7, 14–16^. In addition, σ^32^ stability is primarily controlled by the inner membrane protease FtsH^17, 18^. Recent studies have demonstrated that the interaction between the signal recognition particle (SRP) and σ^32^ is indispensable for σ^32^ localization at the cell membrane^19, 20^. It is widely accepted that a negative feedback loop exists such that HSR chaperones and proteases titrate free σ^32^ by binding or degrading unfolded proteins, meanwhile the up-regulated σ^32^ increases the transcription of HSPs which subsequently decrease the σ^32^ activity and stability, thus facilitating *E*. *coli* cell viability and proliferation under heat stress^8^.

In addition to endogenous HSPs, the heterologous expression of eukaryotic molecular chaperones increases *E*. *coli* cell viability at high temperatures^21–24^. There is extensive support for the enhanced thermotolerance of transformed *E*. *coli* cells expressing plant small HSPs (sHSPs; 12–43 kDa), such as Oshsp16.9^21^, CsHSP17.5^22^, and RcHSP17.8^23^. Recent studies have shown that expression of CeHSP17, a *Caenorhabditis elegans* sHSP, enables *E*. *coli* cell survival at lethal temperatures^24, 25^. In addition, the introduction of plant late embryogenesis abundant proteins^26^ and human disulfide-isomerase^27^ confers protection against heat stress to *E*. *coli* cells. Although the thermoprotective properties of various exogenous proteins have been extensively reported, the acquired thermotolerance is largely attributed to their conserved chaperone functions, raising the question of whether other types of eukaryotic proteins have similar protective effects.

Here, we report that heterologous expression of a RING (Really Interesting New Gene) domain E3 ligase from *Brassica napus*, named BnTR1, conferred pronounced thermoprotection on *E*. *coli* cells. BnTR1 dramatically increased the expression of numerous *E*. *coli* HSPs under both normal and heat stress conditions. Further experiments revealed that BnTR1 expression induced the accumulation of heat shock factor σ^32^. However, unlike molecular chaperones such as sHSPs, the RING domain of BnTR1 was the active site for its function in *E*. *coli*. We found that two zinc fingers in the RING domain were able to interact with DnaK and σ^32^, respectively, resulting in σ^32^ stabilization. Together, our findings reveal that heterologous expression of BnTR1 provides thermoprotective effects on *E*. *coli* cells, and it may yield useful insights into the development of engineered thermophilic bacteria.

## Results

### Heterologous expression of BnTR1 enhances *Escherichia coli* thermotolerance and up-regulates HSPs

**Figure 1:**
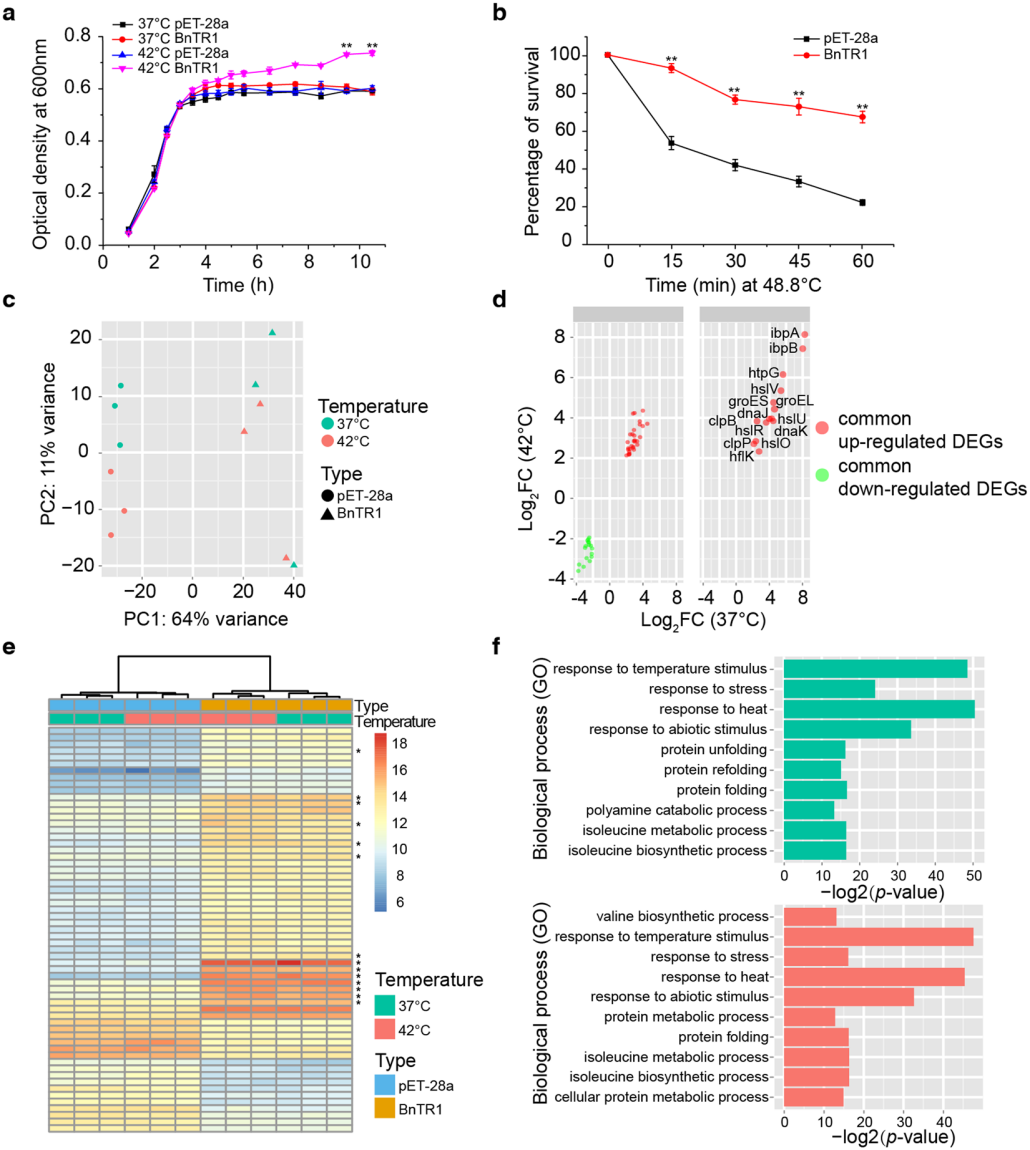
Phenotypic characterization and transcriptional changes of *E*. *coli* cells expressing BnTR1. (**a**) Growth curves of pET and pET-BnTR1 cells at 37 °C and 42 °C. The two-tailed Student’s *t*-test was used to compare the growth rates of cells cultured at 42 °C (**p-value < 0.001). (**b**) Survivability at 48.8 °C. The two-tailed proportion test was applied for the comparison (**p-value < 0.001). (**c**) Transcriptome patterns of pET and pET-BnTR1 cells cultured at normal and high temperatures were determined by PCA. (**d**) Distribution of 61 common DEGs by comparing pET-BnTR1 with pET cells cultured at 37 °C (x-axis) and 42 °C (y-axis). Axes are denoted with a log_2_-transformed fold-change (FC). The 14 significantly up-regulated heat shock genes are separated (right panel). (**e**) Heatmap of common DEGs. The pET and pET-BnTR1 cells and two culture conditions are identified using different colours. HSPs were marked with asterisks. (**f**) Top 10 significant GO terms in pET-BnTR1 cells are at 37 °C (top) and 42 °C (bottom). The x-axis denotes the negative log_2_-transformed *p*-value. The data are presented as means ± s.d. from three independent experiments (**a**,**b**).

Our previous study demonstrated that BnTR1 plays a key role in conferring thermal resistance among multiple plant species^28^. Surprisingly, we observed a similar trend when we expressed BnTR1 in *E*. *coli*. There was little change in growth rates between pET and pET-*BnTR1* cells at the normal temperature (Fig. 1a), while transformed cells expressing BnTR1 showed superior growth over cells expressing the empty vector alone upon temperature up-shift. After 10 hours of heat stress, pET-*BnTR1* cell growth was significantly greater than the total pET cell growth (Fig. 1a). Noticeably, after 1 hour of exposure at 48.8 °C, 67% of pET-*BnTR1* cells survived, while only 22% of cells with the empty vector survived (Fig. 1b). Hence, heterologous expression of BnTR1 provided *E*. *coli* cells with tolerance against heat stress without affecting growth under normal culture conditions.

To further assess the impact of BnTR1 expression, we performed microarray analyses to explore the transcriptional changes of *E*. *coli* cells when cultured at 37 °C or 42 °C. Principal component analysis (PCA) was first applied to determine the distance between the transcriptomes (Fig. 1c). The first principal component (PC1) holding the largest variance (64%) distinctly clustered pET-*BnTR1* cells and pET cells into two groups. We also noted that the second principal component (PC2) contributed 11% variance and slightly separated the samples by culture temperatures. These data demonstrated that changes to the transcriptome were primarily due to BnTR1 expression.

Next, to achieve a robust list of differentially expressed genes (DEGs), we used five independent statistical methods with stringent thresholds (Supplementary Fig. S1a). In consequence, we found that BnTR1 altered the expression levels of 112 and 122 genes at 37 °C and 42 °C, respectively (Supplementary Tables S1 and S2). Intriguingly, nearly half (44 up-regulated and 17 down-regulated) of all DEGs were detected under both normal and heat stress conditions (Supplementary Fig. S1b), suggesting that BnTR1 expression induced conserved transcriptional changes at different temperatures. In particular, many bacterial HSPs were significantly up-regulated upon BnTR1 expression (Fig. 1d,e). Specifically, expression of the DnaK/DnaJ and GroEL/GroES chaperone teams, which function to re-fold and stabilize denatured proteins^29^, increased 16- and 24-fold in pET-*BnTR1* cells compared with cells expressing the empty vector (Supplementary Table S3). Furthermore, the levels of proteases, such as HslU/HslV, which function in protein degradation, were approximately 14-fold higher in pET-*BnTR1* cells (Fig. 1d). To explore the physiological functions of DEGs, we performed gene ontology (GO) analysis. Consistent with the increase in HSPs, the most significantly changed GO terms were closely related to “response to heat” and “protein folding” processes (Fig. 1f). To determine whether the transcriptome changes were due to the stress of over-expression proteins, we set a control group with *E*. *coli* cells expressing PUB18, which is a U-box E3 ligase from *Arabidopsis thaliana* ^30^. We did not observer significant changes of the HSP gene *dnaK* in PUB18 expressing *E*. *coli* cells (Supplementary Fig. S2). Taken together, these data suggest that the heterologous expression of BnTR1 in *E*. *coli* specifically up-regulated bacterial HSPs.

### BnTR1 expression induces σ^32^ accumulation

**Figure 2:**
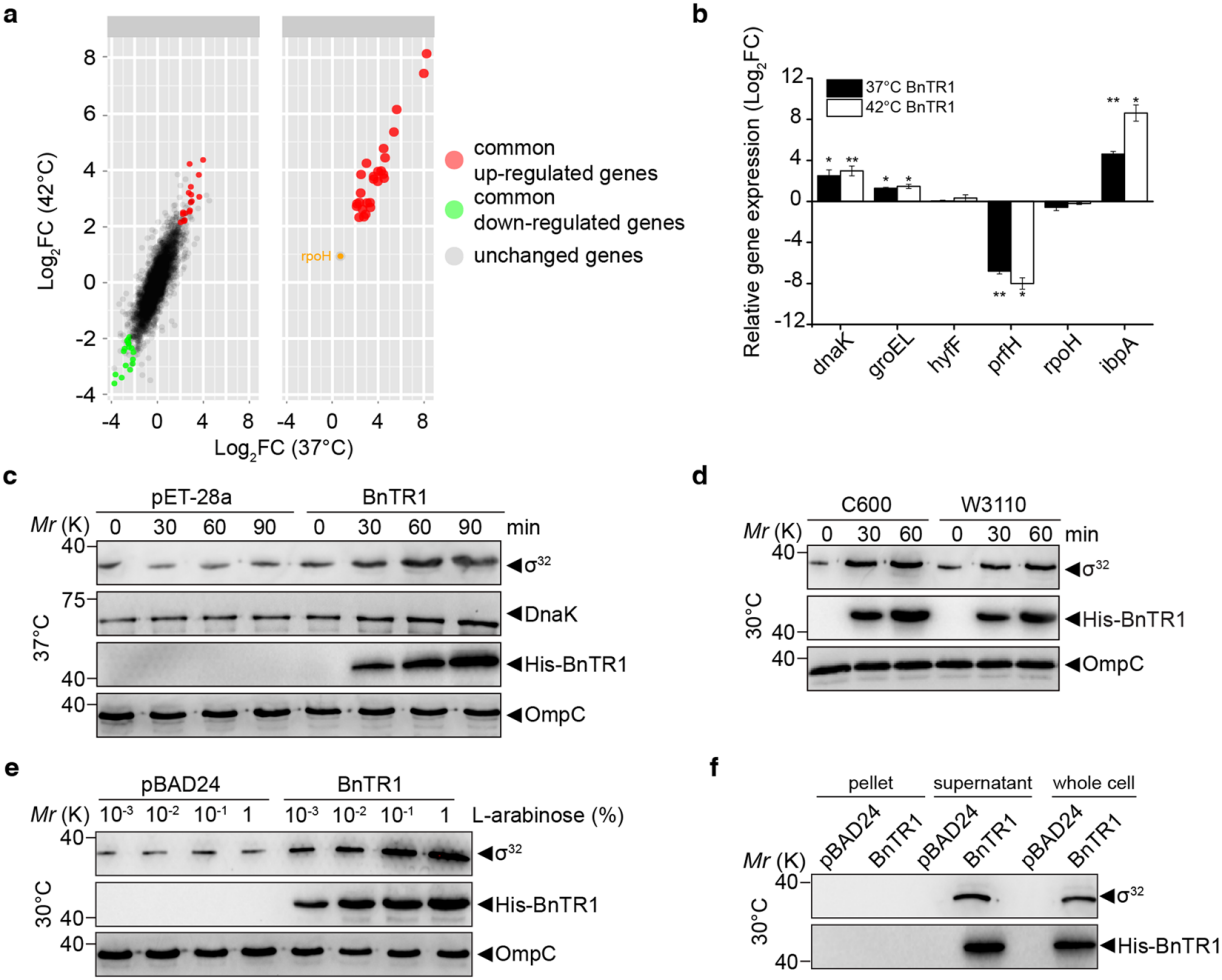
The σ^32^ level is increased during the expression of BnTR1. (**a**) Distribution of σ^32^ regulons among the common DEGs (right panel). The expression level of *rpoH* measured by microarray is denoted as an orange dot. (**b**) Validation of microarray data by *q*RT-PCR and two-tailed Student’s *t*-test was used for the comparison (*p-value < 0.05 and **p-value < 0.001). Six genes were selected, including three heat shock genes (*dnaK*, *groEL*, and *ipbA*), one down-regulated DEG (*prfH*), and two genes without significance (*hyfF* and *rpoH*). (**c**) Whole-cell extracts from pET and pET-BnTR1 cells cultured at 37 °C with 0.1 mM IPTG were analysed using Western blotting (probed with anti-DnaK, anti-σ^32^, and anti-His for BnTR1). (**d**) Wild-type *E*. *coli* W3110 and C600 cells harbouring the pBAD24 empty vector or pBAD-BnTR1 were grown at 30 °C with 0.1% L-arabinose. Whole-cell extracts taken at the indicated time were loaded and probed with anti-σ^32^ and anti-His for BnTR1. **(e)** Whole-cell extracts were from *E*. *coli* C600 cells induced with different concentration of L-arabinose for 30 min at 30 °C. Immunoblots were shown and probed with antibodies indicated. (**f**) Western blotting (probed with anti-σ^32^ and anti-His for BnTR1) of whole cell, supernatant, and pellet proteins that were extracted as described in (**d**). OmpC was detected in parallel by anti-OmpC and used as a loading control in **(c**,**d** and **e)**. The data are presented as means ± s.d. of three independent experiments (**b**). Full-length blots are presented in Supplementary Figure 6.

Because the σ^32^ is the central player in regulating HSP transcription^8^, we next investigated the changes of the σ^32^ level in *E*. *coli* cells expressing BnTR1. Interestingly, more than half of the common up-regulated DEGs, together with HSP genes, were directly regulated by σ^32^ (Fig. 2a and Supplementary Table S3). Three σ^32^ regulons, *dnaK*, *groEL*, and *ipbA*, were selected and validated their up-regulation using quantitative RT-PCR (*q*RT-PCR). Notably, *rpoH* (the gene encoding σ^32^) remained at its basal transcriptional level, which was confirmed by both microarray and *q*RT-PCR (Fig. 2a,b). These data indicate that BnTR1 may not participate in the transcriptional regulation of σ^32^.

Measuring the protein levels further supported the assumption of post-transcriptional regulation of σ^32^. The BnTR1 expression rapidly trigged σ^32^ accumulation within 30 min, and it persisted for at least 90 min (Fig. 2c). In accordance with the increased transcription, DnaK synthesis was concomitantly increased and reached its peak level after 1 hour (Fig. 2c), indicating that cellular σ^32^ was in an active state. Because abnormal protein production in *E*. *coli* can also increase HSPs^31, 32^, we examined whether the increase in σ^32^ was due to the aggregation of unfolded BnTR1. To minimize the basal BnTR1 protein level and to avoid its toxic effect on *E*. *coli* cells, *BnTR1* with a tightly regulated pBAD promoter was generated and induced by L-arabinose under a low temperature in *E*. *coli* W3110 and C600 strains. Remarkably, σ^32^ levels still increased dramatically even when BnTR1 was slightly induced at 30 °C (Fig. 2d). We also tested dosage effects of BnTR1 on the σ^32^ accumulation by using different concentrations of inducer. When supplemented with L-arabinose, σ^32^ levels increased in *E*. *coli* expressing BnTR1 compared with the control groups harbouring empty vectors. Moreover, when BnTR1 was induced, both the σ^32^ level and the amount of BnTR1 were higher in *E*. *coli* cells treated with 1% L-arabinose than with 0.01% and 0.001% L-arabinose, respectively (Fig. 2e). In addition, cell lysate tests demonstrated that the majority of BnTR1 was concentrated in the supernatant and was barely detectable in the inclusion bodies (Fig. 2f).

### The RING domain is the active site of BnTR1

**Figure 3:**
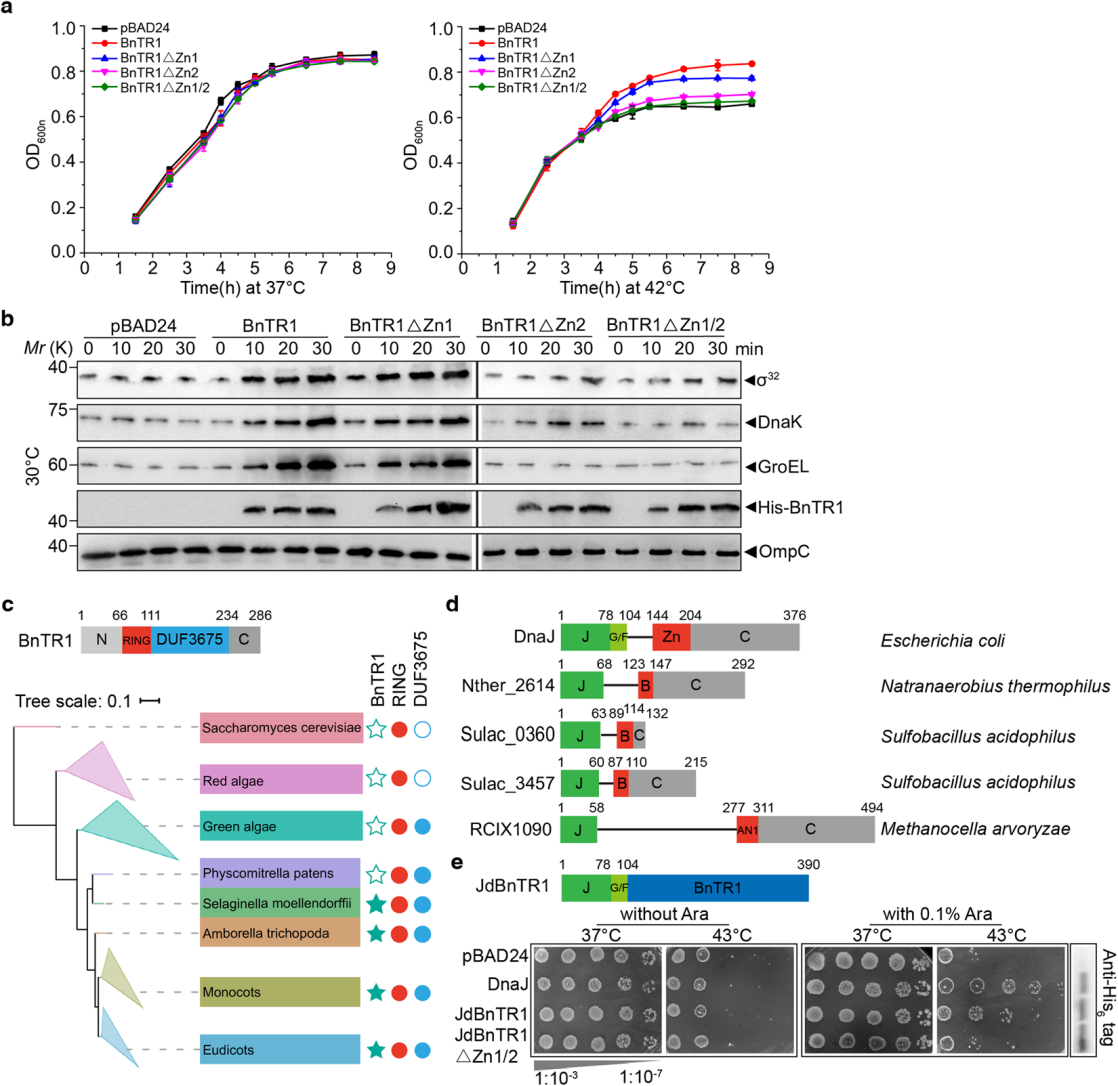
Two zinc fingers of BnTR1 have distinct functions. (**a**) Growth curves of *E*. *coli* C600 cells with BnTR1 mutants at 37 °C (left) and 42 °C (right). (**b**) Western blot with antibodies against His-BnTR1 (His antibody), σ^32^ and chaperones (DnaK and GroEL). Whole-cell extracts were obtained from wild-type *E*. *coli* C600 cells. OmpC was used a loading control. Samples between gels were from the same experiments and were processed in parallel. (**c**) Diagrams indicate the BnTR1 domain structure. A reconstructed phylogenetic tree of *Plantae*, in which clades with multiple species (red algae, green algae, monocots, and eudicots) are collapsed. The present and absent homologues of full-length BnTR1, RING domain, and DUF3675 domain are denoted with filled and empty shapes, respectively. Drawn to scale: amino-terminus (N), DUF3675 (unknown function domain closely associated with zinc finger) domain and carboxyl-terminus (C). (**d**) Domain structures of representative prokaryotic proteins containing both J-domains and zinc fingers. Drawn to scale: zinc finger (Zn), J (J-domain), G/F (glycine/phenylalanine-rich) and B (B-box). (**e**) Complementation of temperature sensitive *E*. *coli* stains expressing pBAD24 constructed DnaJ, chimeric BnTR1 and mutants at 43 °C. The data are presented as means ± s.d. of three independent experiments (**a**). Full-length blots are presented in Supplementary Figure 6.

As an E3 ligase, BnTR1 possesses a typical RING domain chelating two zinc atoms to form two zinc fingers^28^, which led us to explore whether the RING domain is essential for BnTR1 function in bacteria. The BnTR1 mutants, BnTR1ΔZn1 (BnTR1^C66S/C69S^), BnTR1ΔZn2 (BnTR1^C82S/C84S^) and BnTR1ΔZn1/2 (BnTR1^C66S/C69S/C82S/C84S^), had no influence on cell growth at the normal temperature (Fig. 3a). As expected, wild-type BnTR1 kept the *in vitro* E3 ubiquitin ligase activity, and *E*. *coli* cells benefited from the expression of wild-type BnTR1 at 42 °C (Fig. 3a and Supplementary Fig. S3). Interestingly, though BnTR1 mutants lost the ligase activity, they had different effects on *E*. *coli* cells. The growth rate of BnTR1ΔZn1 cells was similar but slightly lower than that of BnTR1 cells (Fig. 3a). In sharp contrast, the growth of BnTR1ΔZn2 and BnTR1ΔZn1/2 cells significantly decreased (Fig. 3a). The cell growth results indicated that mutations in the first zinc finger (Zn1) did not substantially affect BnTR1 activity, but the second zinc finger (Zn2) was more crucial.

To identify the distinct functions of the two zinc fingers, the levels of HSPs and σ^32^ were measured. HSPs in BnTR1ΔZn1/2 and BnTR1ΔZn2 cells dropped to basal levels; however, mutation of Zn1 had little effect on HSPs as the HSP levels were similar in BnTR1ΔZn1 and wild-type BnTR1 cells. We observed a positive correlation between σ^32^ levels and HSP synthesis. The expression of wild-type BnTR1 and BnTR1ΔZn1 induced the accumulation of σ^32^ over 10 to 30 min, whereas no significant changes of σ^32^ levels were detected in BnTR1ΔZn1/2 and BnTR1ΔZn2 cells (Fig. 3b). It is important to note that the cells expressing mutant BnTR1 were cultured under non-stress conditions (30 °C). These results were in strong agreement with the growth rate experiments, suggesting that zinc fingers play an important role in the function of BnTR1; in particular, Zn2 was indispensable for the up-regulation of HSPs and σ^32^.

To further understand BnTR1 activity in *E*. *coli*, we next explored the distribution of its homologues. By mapping BnTR1 homologues to the reconstructed phylogenetic tree, we found that BnTR1 homologues emerged in ferns (*Selaginella moellendorffii*) but are missing in mosses (*Physcomitrella patens*), green algae, and red algae. Interestingly, unlike full-length BnTR1, the RING domain was widely spread among the *Plantae* and was even detected in yeast (*Saccharomyces cerevisiae*) (Fig. 3c and Supplementary Fig. S4a). Considering the importance of the RING domain, we next conducted a domain search in prokaryotic genomes. In *E*. *coli*, DnaJ chaperone contained a zinc-finger domain next to the J-domain^33^. Moreover, other types of zinc fingers, such as the B-box and AN1, were integrated with the J-domain in specific bacterial and archaeal species^34^ (Fig. 3d), raising the possibility that BnTR1 and DnaJ are analogous in terms of their structural topology or enzymatic activity. The comparison of the structures of BnTR1 and DnaJ by molecular modelling indicated that the eight residues (cysteine/histidine) of BnTR1 coordinated the zinc ions in a cross-braced shape (Supplementary Fig. S4b). By contrast, the two zinc fingers in DnaJ constituted a right angle and formed a V-shaped molecule^33^ (Supplementary Fig. S4c).

The dissimilar topology led us to extend the comparison of zinc fingers *in vivo*. A complementary experiment using the *dnaJ* mutant *E*. *coli* strain MF634 revealed that cells expressing wild-type BnTR1 formed very few clones at 43 °C, indicating that BnTR1 alone was unable to compensate for the loss of DnaJ (Supplementary Fig. S5). Because the J-domain and glycine/phenylalanine-rich (G/F) region are crucial for DnaJ chaperone functions^35^, a chimeric protein made by concatenating BnTR1 with the J-domain and G/F region of DnaJ was engineered and named JdBnTR1 (Fig. 3e). Surprisingly, JdBnTR1 rescued the defective cells at the high temperature, but mutations in both zinc fingers (JdBnTR1ΔZn1/2) were incapable of rescuing the growth defect at 43 °C (Fig. 3e). Taken together, these results suggest that the up-regulation of HSPs and σ^32^ was not a result of over-expression of foreign proteins or possible BnTR1 solubility problems, but rather the physiological function of BnTR1 in the post-transcriptional regulation of σ^32^.

### BnTR1 stabilizes σ^32^*in vivo* through its RING domain

**Figure 4:**
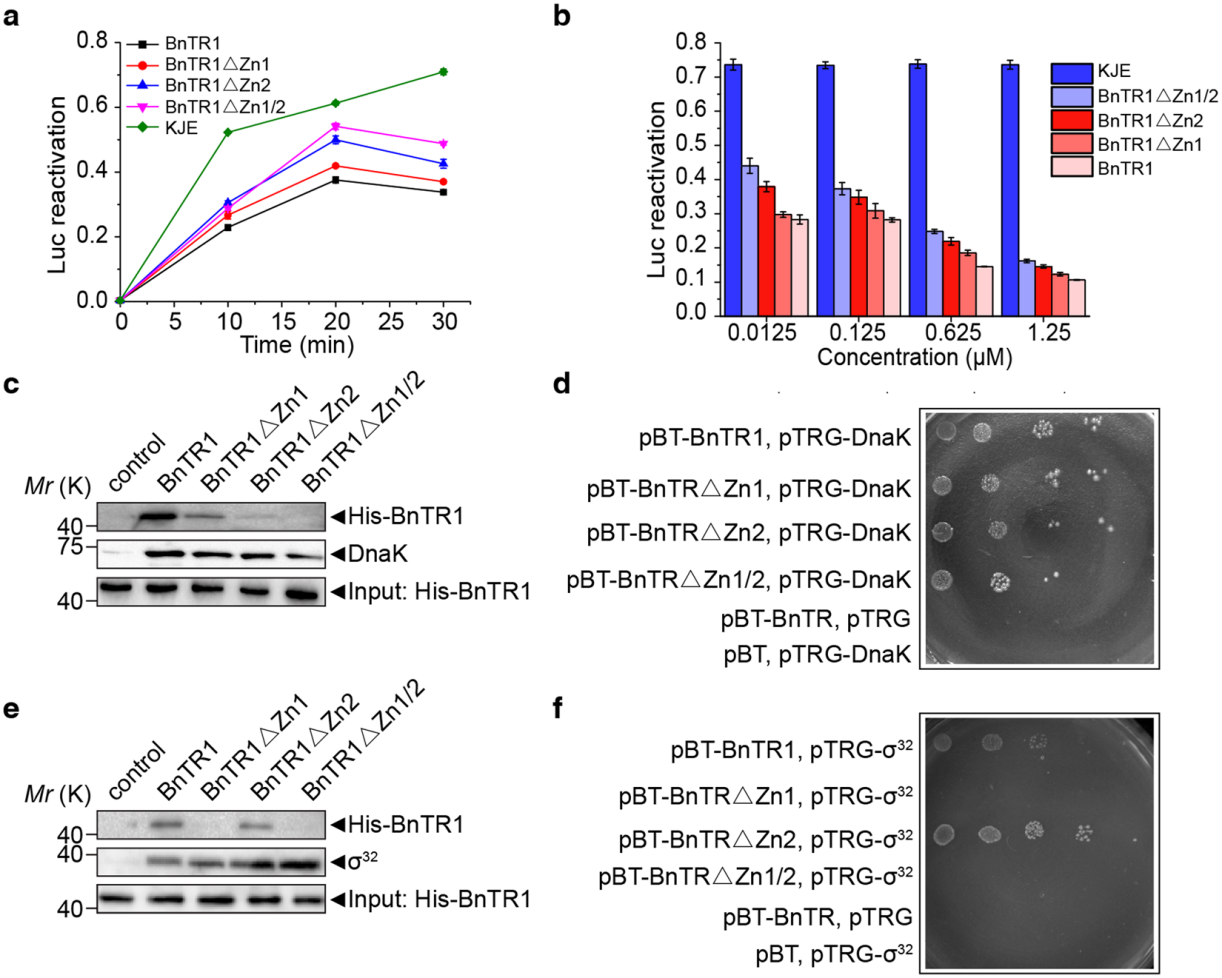
BnTR1 interacts with DnaK and σ^32^ *in vivo*. (**a**) Refolding denatured luciferase by the KJE chaperone team with or without BnTR1 or BnTR1 mutants (0.0625 μM). (**b**) The same reactivation system as described in (**a**) was used with a series of BnTR1 or BnTR1 mutant concentrations incubated for 30 min. (**c**) and (**e**) Co-immunoprecipitation of BnTR1 with DnaK (**c**) and σ^32^ (**e**). Purified BnTR1 and mutant forms were incubated with DnaK or His-σ^32^. The control group did not contain DnaK or His-σ^32^, and the input lane was used to adjust the concentration of BnTR1 and mutant forms in the reaction system. (**d**) and (**f**) Interaction analysis of BnTR1 and DnaK (**d**) or σ^32^ (**f**) in the bacterial two-hybrid system. The data are presented as means ± s.d. of three independent experiments (**a**,**b**). Full-length blots are presented in Supplementary Figure 6.

Because σ^32^ activity is negatively controlled by the KJE chaperone team^15, 36^, it is unknown whether BnTR1 could hinder KJE-dependent regulation of σ^32^. The assay of KJE refolding of denatured luciferase substrate was employed to test the function of two zinc fingers of BnTR1. BnTR1 and its mutant forms sharply decreased the reactivation activity of the KJE chaperon team in 10 minutes (Fig. 4a). However, along with increasing the reaction time, BnTR1 mutants displayed different effects. After 20 min, BnTR1ΔZn2 and BnTR1ΔZn1/2 showed distinguishably higher levels of reactivated luciferase than the wild-type BnTR1 and BnTR1ΔZn1. To confirm our observations, series of BnTR1 mutant concentrations were supplied into the reaction. We confirmed the inhibitory effects, which were even more significant when we used a low concentration (0.0125 μm) of BnTR1 mutant (Fig. 4b). Based on these results, BnTR1 could act as an intruder in the KJE refolding system *in vitro* and confirmed the distinct functions of two zinc fingers.

As the Zn2 of BnTR1 is more important for its inhibitory effect, we hypothesized that this region physically interacts with the DnaK chaperone. Therefore, 6His-BnTR1 (N terminus) and BnTR1 mutants were purified and used as prey in co-immunoprecipitation (Co-IP) experiments. Indeed, DnaK and BnTR1 formed a stable complex, which remained intact when Zn1 was mutated (BnTR1ΔZn1) (Fig. 4c). As expected, the DnaK-BnTR1ΔZn2 or DnaK-BnTR1ΔZn1/2 complex was barely detectable, suggesting that DnaK interacted with the Zn2 of BnTR1 *in vivo*. We next used a bacterial two-hybrid system to confirm their interaction. We identified a clear signal for the DnaK-BnTR1 and DnaK-BnTR1ΔZn1 interactions (Fig. 4d).

It is known that σ^32^ is a substrate of DnaK and the co-chaperone DnaJ^36^. We observed different growth rates (Fig. 3a) and inhibition effects (Fig. 4a) between BnTR1ΔZn1 and BnTR1ΔZn1/2, suggesting that Zn1 may play a minor role in the stabilization of σ^32^. Surprisingly, BnTR1 and σ^32^ formed a rather tight complex, and mutation of Zn2 had no effect on their interaction (Fig. 4e). The interaction between BnTR1 and σ^32^ was dependent on Zn1, as mutation in this region completely disrupted the interaction, which was demonstrated by Co-IP and bacterial two-hybrid experiments (Fig. 4e,f).

## Discussion

In this study, we report heterologous expression BnTR1, a plant E3 ligase containing a RING domain, could effectively protect *E*. *coli* cells from heat stress. BnTR1 expression in *E*. *coli* dramatically increased the level of heat shock factor σ^32^, even at low temperatures, resulting in the significant up-regulation of HSPs. It has been well established that HSP expression rapidly increases following temperature upshift to protect *E*. *coli* cells from heat-damaged proteins^1, 3, 4^. In our study, several HSPs were induced by BnTR1 expression, including DnaK, GroEL, ClpB and HtpG, which are molecular chaperones with functions in deterring unfolded protein aggregation and assisting in their refolding^29, 37^. Other HSPs, such as HslU/hslV and ClpP, are proteases that function to degrade and dissolve heat-denatured proteins^38^. Thus, the increased heat resistance largely depends on the cumulative effects of multiple molecular chaperones and proteases, which are essential to refold and degrade heat-damaged proteins. Therefore, cells expressing BnTR1 seem less affected and damaged by heat stress. Consistent with this idea is a recent report that over-expression of the GroEL/GroES chaperones increase the maximum growth temperature of wild-type *E*. *coli* to 47.5 °C^39^. Heat shock factor σ^32^ is known to be the key regulator of *E*. *coli* HSPs^5^. Although BnTR1 triggered considerable activation of σ^32^, which is sufficient to induce HSPs, σ^32^ transcription remained relatively constant. Thus, the apparent linkage between BnTR1 and σ^32^ is at the post-transcriptional level.

It could be argued that the enhanced HSP levels are not due to a specific BnTR1 function, as previous studies have shown that overexpression of abnormal proteins increases HSP levels^31, 32^. The simplest explanation for the induction of HSPs is the accumulation of unfolded BnTR1. However, this simple model seems inconsistent with our current data. These reported abnormal proteins are misfolded or unfolded with aberrant high-order structure, and are found in inclusion fractions^31, 32^, while the majority of BnTR1 protein was soluble. In addition, BnTR1 in *E*. *coli* remained active rather than being unfolded. Thus, the thermotolerance of *E*. *coli* was not due to the stress of the over-expression of exogenous proteins.

**Figure 5:**
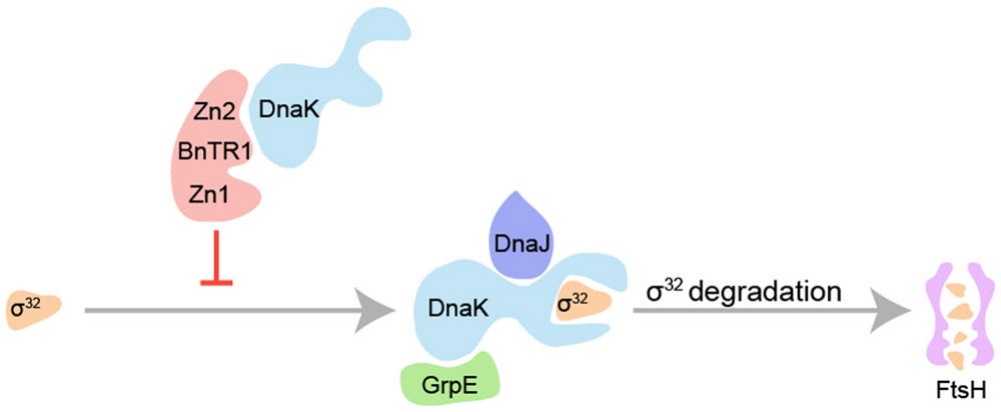
Proposed model of σ^32^ accumulation in *E*. *coli* cells expressing BnTR1. In wild-type *E*. *coli* cells, the KJE chaperone team interacts with σ^32^ to inhibit its activity, and then facilities σ^32^ degradation by the protease FtsH. In *E*. *coli* cells expressing BnTR1, the Zn2 in its RING domain of BnTR1 may interact with DnaK, thereby stabilizing σ^32^.

The heterologous expression of eukaryotic molecular chaperones enables *E*. *coli* cells to resist heat stress at lethal temperatures^21–24, 27^. This protective effect is closely related to chaperone activity in maintaining proteins in a folded state or preventing unfolded proteins from aggregation^25–27^. However, this is not the case for BnTR1, as purified BnTR1 exhibited no chaperone function in refolding denatured luciferases with KJE *in vitro*; instead, the efficiency of BnTR1 largely depended on its RING domain. Thus, we propose an alternative model. The KJE chaperone team inhibits the σ^32^ activity by directly binding to σ^32^ *in vivo* and *in vitro* ^14–16^. BnTR1 interacts with DnaK to form a stable complex mainly through the Zn2, which may inhibit the negative effect of KJE on σ^32^, resulting in the accumulation of active σ^32^. Moreover, although mutation in Zn1 had little effect on σ^32^ and HSP levels, our *in vivo* interaction studies suggest that Zn1 selectively interacts with σ^32^. It is not clear whether Zn1 and/or Zn2 could influence pathways associated with SRP and the protease FtsH, key regulators for σ^32^ membrane localization and degradation^17–20^. Thus, we suggest that the increased amount of active σ^32^ is largely attributed to the Zn2 of BnTR1 (Fig. 5).

BnTR1 is identified as a key player in the heat stress response of *B*. *napus*, and constitutive *BnTR1* expression significantly increases the heat tolerance of multiple plant species^28^. Interestingly, we observed that heterologous expression of BnTR1 also conferred pronounced thermoprotective effects on *E*. *coli* cells. In plant the function of BnTR1 tightly relies on its E3 ligase activity, but BnTR1 may work in a different way in *E*. *coli*. All BnTR1 mutants used in this study lost their *in vitro* E3 ligase activity, whereas the effect of BnTR1ΔZn1 in *E*. *coli* was slightly affected. Although no significant homologues of BnTR1 have been detected in prokaryotes, we noted that the *E*. *coli* chaperone DnaJ possesses a domain containing two zinc fingers. Importantly, analogous to BnTR1, the two DnaJ zinc fingers play different roles such that one is important for DnaK-independent chaperone activity, while the other is essential for the interaction with DnaK^40, 41^. A rather surprising finding was that the expression of the JdBnTR1 chimeric protein (a fusion of the J-domain and BnTR1) rescued the *dnaJ* mutant strains at a high temperature. Thus, it suggested that BnTR1 and other proteins possessing similar zinc figure domains may be useful resources in the genetic engineering of thermophilic bacteria for industrial application.

## Methods

Further details of bacterial viability assays, two-hybrid assays, recombinant protein expression and purification, *in vitro* assay of E3 ubiquitin ligase activity, Co-IP assays, and RNA extraction and *q*RT-PCR can be found in the Supplementary Methods.

### Strains, plasmids and growth condition

The *E*. *coli* strains and plasmids were commercially obtained from the American Type Culture Collection (ATCC) and the Coli Genetic Stock Center (CGSC) (Supplementary Table S4). The strains and their transformants were grown aerobically in Luria–Bertani (LB) medium (tryptone 10 g L^−1^, NaCl 10 g L^−1^ and yeast extract 5 g L^−1^, pH 7.4) and were supplemented with ampicillin (100 μg mL^−1^) and kanamycin (50 μg mL^−1^) when necessary. To validate the *in vitro* interaction of DnaK and σ^32^ with BnTR1, the expression of recombinant proteins from *E*. *coli* Rosetta (DE3) was induced at the mid-logarithmic phase (OD_600nm_ = 0.6) using 0.5 mM isopropyl β-D-1-thiogalactopyranoside (IPTG) for 30 min.

### Vectors construction

pET-28a and pBAD24 vectors were used to express polyhistidine (6His)-tagged proteins (Supplementary Table S5). The construct expressing 6His-BnTR1 (N-terminal) was generated as follows. *BnTR1* and *PUB18* (AT1G10560) was amplified by PCR from *B*. *napus* and *A*. *thaliana*, respectively, and ligated into pET-28a (digested with BamHI and HindIII). 6His-*BnTR1* was then sub-cloned into the pBAD24 vector (digested with NcoI and HindIII) to obtain pBAD24-*BnTR1*. Site-specific mutations were introduced into BnTR1 using QuikChange® Lightning Site-Directed Mutagenesis Kits (Stratagene) with the resultant plasmid pET-28a-*BnTR1* as the template. The constructs containing mutant BnTR1 (pET-28a-/pBAD24-*BnTR1* ^C66S/C69S^, pET-28a-/pBAD24-*BnTR1* ^C82S/C84S^ and pET-28a-/pBAD24-*BnTR1* ^C66S/C69S/C82S/C84S^) were transformed into *E*. *coli* and induced to express the target proteins.

The 6His-*dnaJ* was amplified by using *E*. *coli* C600 as the template and ligated into pET-28a (digested by BamHI and Xhol). pBAD24-*dnaJ* was then constructed by subcloning of the Ncol/Xhol *dnaJ* fragment from pET-28a-*dnaJ*. For the construction of pBAD24-*JdBnTR1*, the J domain of *dnaJ* (312 bp) was amplified from pET-28a-*dnaJ* and then ligated into pBAD24-*BnTR1* (digested by NdeI and BamHI). The gene encoding for σ^32^ (*rpoH*) was obtained by amplifying its entire coding region (855 bp) from *E*. *coli* C600 and then cloned into pET-28a (digested by BamHI and XhoI) to get the pET-28a-*rpoH*. To create pET-28a-*luc*, the luciferase gene was amplified and ligated into pET-28a (digest by BamHI and XhoI). Successful transformants were analyzed by colony PCR and constructs containing correct inserts were sequenced to ensure the accuracy.

### Luciferase refolding assays

Luciferase refolding was evaluated as described previously with modifications^42^. Specifically, luciferase (25 μM) was denatured and diluted 100-fold into a premix containing 10 mM MOPS (pH 7.2), 50 mM KCl, 5 mM MgCl_2_, 0.015% (w v^−1^) bovine serum albumin (BSA), 0.1 mg ml^−1^ creatine kinase, 20 mM creatine phosphate, 5 mM ATP, 2 μM DnaK, 0.5 μM DnaJ, 0.125 μM GrpE, and a serial concentration of BnTR1. Luciferase activity was continuously monitored at 22 °C using a luciferase assay system from Molecular Devices (L MaxII^384^).

### Immunoblotting

Western blotting was employed to determine the translational level of σ^32^ and heat shock proteins. Briefly, *E*. *coli* C600 and W3110 pBAD24 transformants (OD_600nm_ = 0.6) were induced with 0.1% L-arabinose for 30 minutes. Cultures were harvested and divided into two equal aliquots. One aliquot was immediately precipitated with 5% trichloracetic acid (TCA) for protein concentration quantification. The precipitate was collected by centrifugation and washed with 80% iced acetone. Pellets were dried under vacuum and re-suspended with double distilled water. The concentration of proteins was quantified using pierce BCA protein assay kit (Thermo). The other aliquot was collected and re-suspended in SDS-PAGE sample buffer for western blotting analysis.

Proteins were separated by SDS-PAGE and transferred to nitrocellulose membranes (Bio-Rad). Membranes were blocked with 10% bovine serum albumin (BSA) diluted in TBST (TBS and 0.1% (w v^−1^) Tween 20) for 1 hour at room temperature and then incubated overnight at 4 °C with anti-σ^32^ (1:1,000) (Neoclone), anti-DnaK (1:10,000) (Abcam), anti-GroEL (1:1,000) (Abcam), anti-His (1:1,000) (Abcam), and anti-OmpC (1:1,000) (Biorbyt) antibodies (diluted in TBST). Membranes were washed three times with TBST, incubated for 1 hour at room temperature with a 1:1,000 dilution of HRP-conjugated secondary antibodies in TBST, and washed three times with TBST. Immunoreactive bands were detected using Pierce ECL Western Blotting Substrate (Thermo Scientific) and ChemiDoc XRS Plus (Bio-Rad). Images were captured with Image Lab Software version 3.0 (Bio-Rad).

### Phylogenetic and protein domain analysis

We constructed a phylogenetic tree of 51 *Plantae* species according to the method described by Ciccarelli *et al*.^43^. We selected highly conserved proteins without horizontal gene transfers effects, and the list included ribosomal proteins (S2, S3, S4, S5, S7, S8, S11, S12, L3, L5, L11, L9E/L22, L10E, and L15), aminoacyl tRNA synthetases (Seryl-, Phenylalanine-, and Leucyl-tRNA synthetase), metal-dependent proteases with chaperone activity, and predicted GTPase probable translation factor. The MUSCLE program^44^ was used to conduct multiple sequence alignments with iteration number set to 100, and then aligned sequences were concatenated. The phylogenetic tree was constructed using RAxML^45^ (parameters: -f a -T 6 -m PROTGAMMAJTTX -p -x -#autoMRE). *Saccharomyces cerevisiae* was set as the outgroup.

Significant homologs of full-length BnTR1 was detected by BLASTP (E-value < 10^−17^). The Kyoto Encyclopedia of Genes and Genomes (KEGG) database^46^ was used to construct the distribution of J-domain, RING, AN1, and B-box protein domains (E-value < 10^−4^).

### Microarray procedures

Cy3 fluorescently-labeled cRNA was prepared and hybridized to Agilent *E*. *coli* whole-genome gene expression microarrays (8*15 K) according to the single channel microarray-based protocol (Agilent Technologies Inc.). The biological repeats were randomly distributed onto two microarray slides. The array images were scanned by the G2565BA Microarray Scanner System, and raw data were then normalized (quantile method), merged and filtered by Feature Extraction Software (Agilent Technologies Inc.).

The control probes and probes without annotation were dropped out as they were considered to have non-transcriptome biological meanings. As the Agilent *E*. *coli* microarray contains three other types of probes (designed for *E*. *coli* O157:H7, CFT073 and EDL933), these probes were neglected before the downstream analysis. For the “sibling probe-set”, one gene corresponding to multiple probes, the average value to represent the gene expression intensity^47^. A total of 4108 genes were finally selected, the intensity values were log2 transformed.

To identify robust DEGs, five parametric and non-parametric methods were independently applied. First, three traditional processing methods, the Student’s *t*-test, the Mann-Whitney *U*-test, and fold-change calculation were applied. The DEGs in each comparison were identified with the false discovery rate (FDR) <0.05 for the *t*-test and *U*-test, together with fold-change cut-off values of 2.0-fold decrease and increase. Two more sophisticated methods in R/Bioconductor packages “Limma”^48^ and “RankProd”^49^ were used (Supplementary Table S6). The intersection of DEGs identified using the five methods were considered to be significantly up- or down-regulated in BnTR1 strains at 37 °C and 42 °C, respectively. The σ^32^ regulons were summarized from Nonaka *et al*.^50^.

The latest GO information (submission date: 7/1/2015) of *E*. *coli* was retrieved from Gene Ontology Consortium. The minimum number of genes in each GO term was set as 5. The Fisher’s exact test was used to determine the significant GO terms with threshold of *p*-value < 0.05.

For microarray data validation, the transcriptional levels of genes were detected (Supplementary Table S7). RNA was extracted as described above, cDNA was generated using an iScript cDNA synthesis kit (Bio-Rad) and *qPCR* was performed using SYBR Green Supermix (Bio-Rad). The results were analysed, and the mRNA levels were normalized against that of 16 s rRNA using the ΔΔ*CT* method^51, 52^.

### Statistical analysis

All statistical analysis was conducted by the R software.

### Data availability

The microarray data has been deposited in the Gene Expression Omnibus with the accession number GSE85807.

## Electronic supplementary material

Supplementary Information

Supplementary Table 1, Supplementary Table 2, Supplementary Table 3, Supplementary Table 4, Supplementary Table 5, Supplementary Table 6, Supplementary Table 7

## References

[1] Lindquist S (1986). The heat-shock response. Annu Rev Biochem.

[2] Morimoto RI (2011). The heat shock response: systems biology of proteotoxic stress in aging and disease. Cold Spring Harb Symp Quant Biol.

[3] Craig EA, Gambill BD, Nelson RJ (1993). Heat shock proteins: molecular chaperones of protein biogenesis. Microbiol Rev.

[4] Richter K, Haslbeck M, Buchner J (2010). The heat shock response: life on the verge of death. Mol Cell.

[5] Grossman AD, Erickson JW, Gross CA (1984). The htpR gene product of *E*. *coli* is a sigma factor for heat-shock promoters. Cell.

[6] Straus DB, Walter WA, Gross CA (1987). The heat shock response of *E*. *coli* is regulated by changes in the concentration of sigma 32. Nature.

[7] Straus DB, Walter WA, Gross CA (1989). The activity of sigma 32 is reduced under conditions of excess heat shock protein production in *Escherichia coli*. Genes Dev.

[8] Guisbert E, Yura T, Rhodius VA, Gross CA (2008). Convergence of molecular, modeling, and systems approaches for an understanding of the *Escherichia coli* heat shock response. Microbiol Mol Biol Rev.

[9] Guo MS, Gross CA (2014). Stress-induced remodeling of the bacterial proteome. Curr Biol.

[10] Yuzawa H, Nagai H, Mori H, Yura T (1993). Heat induction of sigma 32 synthesis mediated by mRNA secondary structure: a primary step of the heat shock response in *Escherichia coli*. Nucleic Acids Res.

[11] Morita MT (1999). Translational induction of heat shock transcription factor sigma32: evidence for a built-in RNA thermosensor. Genes Dev.

[12] Morita MT, Kanemori M, Yanagi H, Yura T (2000). Dynamic interplay between antagonistic pathways controlling the sigma 32 level in *Escherichia coli*. Proc Natl Acad Sci USA.

[13] El-Samad H, Kurata H, Doyle JC, Gross CA, Khammash M (2005). Surviving heat shock: control strategies for robustness and performance. Proc Natl Acad Sci USA.

[14] Liberek K, Georgopoulos C (1993). Autoregulation of the *Escherichia coli* heat shock response by the DnaK and DnaJ heat shock proteins. Proc Natl Acad Sci USA.

[15] Gamer J (1996). A cycle of binding and release of the DnaK, DnaJ and GrpE chaperones regulates activity of the *Escherichia coli* heat shock transcription factor sigma32. EMBO J.

[16] Guisbert E, Herman C, Lu CZ, Gross CA (2004). A chaperone network controls the heat shock response in *E*. *coli*. Genes Dev.

[17] Herman C, Thevenet D, D’Ari R, Bouloc P (1995). Degradation of sigma 32, the heat shock regulator in *Escherichia coli*, is governed by HflB. Proc Natl Acad Sci USA.

[18] Blaszczak A, Georgopoulos C, Liberek K (1999). On the mechanism of FtsH-dependent degradation of the sigma 32 transcriptional regulator of *Escherichia coli* and the role of the Dnak chaperone machine. Mol Microbiol.

[19] Lim B (2013). Heat shock transcription factor sigma32 co-opts the signal recognition particle to regulate protein homeostasis in *E*. *coli*. PLoS Biol.

[20] Miyazaki R (2016). A Novel SRP Recognition Sequence in the Homeostatic Control Region of Heat Shock Transcription Factor sigma32. Sci Rep.

[21] Yeh CH (1997). Expression of a gene encoding a 16.9-kDa heat-shock protein, Oshsp16.9, in *Escherichia coli* enhances thermotolerance. Proc Natl Acad Sci USA.

[22] Soto A (1999). Heterologous expression of a plant small heat-shock protein enhances *Escherichia coli* viability under heat and cold stress. Plant Physiol.

[23] Jiang C (2009). A cytosolic class I small heat shock protein, RcHSP17.8, of Rosa chinensis confers resistance to a variety of stresses to *Escherichia coli*, yeast and Arabidopsis thaliana. Plant Cell Environ.

[24] Ezemaduka AN (2014). A small heat shock protein enables *Escherichia coli* to grow at a lethal temperature of 50 degrees C conceivably by maintaining cell envelope integrity. J Bacteriol.

[25] Zhang K (2015). A novel mechanism for small heat shock proteins to function as molecular chaperones. Sci Rep.

[26] Zhang X (2014). RcLEA, a late embryogenesis abundant protein gene isolated from Rosa chinensis, confers tolerance to *Escherichia coli* and Arabidopsis thaliana and stabilizes enzyme activity under diverse stresses. Plant Mol Biol.

[27] Fu XM, Zhu BT (2010). Human pancreas-specific protein disulfide-isomerase (PDIp) can function as a chaperone independently of its enzymatic activity by forming stable complexes with denatured substrate proteins. Biochem J.

[28] Liu ZB (2014). A novel membrane-bound E3 ubiquitin ligase enhances the thermal resistance in plants. Plant Biotechnol J.

[29] Saibil H (2013). Chaperone machines for protein folding, unfolding and disaggregation. Nat Rev Mol Cell Biol.

[30] Seo DH (2016). Motif of the Arabidopsis U-Box E3 Ligase PUB18 Is Critical for the Negative Regulation of ABA-Mediated Stomatal Movement and Determines Its Ubiquitination Specificity for Exocyst Subunit Exo70B1. Plant Cell.

[31] Parsell DA, Sauer RT (1989). Induction of a heat shock-like response by unfolded protein in *Escherichia coli*: dependence on protein level not protein degradation. Genes Dev.

[32] Kanemori M, Mori H, Yura T (1994). Induction of heat shock proteins by abnormal proteins results from stabilization and not increased synthesis of sigma 32 in *Escherichia coli*. J Bacteriol.

[33] Martinez-Yamout M, Legge GB, Zhang O, Wright PE, Dyson HJ (2000). Solution structure of the cysteine-rich domain of the *Escherichia coli* chaperone protein Dna. J. J Mol Biol.

[34] Burroughs AM, Iyer LM, Aravind L (2011). Functional diversification of the RING finger and other binuclear treble clef domains in prokaryotes and the early evolution of the ubiquitin system. Mol Biosyst.

[35] Perales-Calvo J, Muga A, Moro F (2010). Role of DnaJ G/F-rich domain in conformational recognition and binding of protein substrates. J Biol Chem.

[36] Rodriguez F (2008). Molecular basis for regulation of the heat shock transcription factor sigma32 by the DnaK and DnaJ chaperones. Mol Cell.

[37] Gragerov A (1992). Cooperation of GroEL/GroES and DnaK/DnaJ heat shock proteins in preventing protein misfolding in *Escherichia coli*. Proc Natl Acad Sci USA.

[38] Burton RE, Baker TA, Sauer RT (2005). Nucleotide-dependent substrate recognition by the AAA + HslUV protease. Nat Struct Mol Biol.

[39] Rudolph B, Gebendorfer KM, Buchner J, Winter J (2010). Evolution of *Escherichia coli* for growth at high temperatures. J Biol Chem.

[40] Linke K, Wolfram T, Bussemer J, Jakob U (2003). The roles of the two zinc binding sites in DnaJ. J Biol Chem.

[41] Shi YY, Tang W, Hao SF, Wang CC (2005). Contributions of cysteine residues in Zn2 to zinc fingers and thiol-disulfide oxidoreductase activities of chaperone DnaJ. Biochemistry.

[42] Perrody E (2012). A bacteriophage-encoded J-domain protein interacts with the DnaK/Hsp70 chaperone and stabilizes the heat-shock factor sigma32 of *Escherichia coli*. PLoS Genet.

[43] Ciccarelli FD (2006). Toward automatic reconstruction of a highly resolved tree of life. Science.

[44] Edgar RC (2004). MUSCLE: multiple sequence alignment with high accuracy and high throughput. Nucleic Acids Res.

[45] Stamatakis A (2014). RAxML version 8: a tool for phylogenetic analysis and post-analysis of large phylogenies. Bioinformatics.

[46] Kanehisa M, Sato Y, Kawashima M, Furumichi M, Tanabe M (2016). KEGG as a reference resource for gene and protein annotation. Nucleic Acids Res.

[47] Liu C (2013). Hyperosmotic response of streptococcus mutans: from microscopic physiology to transcriptomic profile. BMC Microbiol.

[48] Ritchie ME (2015). limma powers differential expression analyses for RNA-sequencing and microarray studies. Nucleic Acids Res.

[49] Hong F (2006). RankProd: a bioconductor package for detecting differentially expressed genes in meta-analysis. Bioinformatics.

[50] Nonaka G, Blankschien M, Herman C, Gross CA, Rhodius VA (2006). Regulon and promoter analysis of the *E*. *coli* heat-shock factor, sigma32, reveals a multifaceted cellular response to heat stress. Genes Dev.

[51] Liu C (2015). Cell cycle control, DNA damage repair, and apoptosis-related pathways control pre-ameloblasts differentiation during tooth development. BMC Genomics.

[52] Liu C (2015). Streptococcus mutans copes with heat stress by multiple transcriptional regulons modulating virulence and energy metabolism. Sci Rep.

