# Supplementary material for: A novel plant E3 ligase stabilizes Escherichia coli heat shock factor σ32
Source: Sci Rep. 2017 Jun 22;7:4045. doi: 10.1038/s41598-017-03056-6 (PMC5481349; doi:10.1038/s41598-017-03056-6)
Supplement: Supplementary file 1 — Supplementary Information [file 41598_2017_3056_MOESM1_ESM.doc]

**Supplementary Information**

**A novel plant E3 ligase stabilizes *Escherichia coli* heat shock factor σ^32^**

Yulong Niu^1^, Xibing Xu^1^, Chengcheng Liu^2^, Tao Wang^1^, Ke Liang^1^, Jianmei Wang^1^, Zhibin Liu^1^, Xufeng Li^1^, Yi Yang^1^

^1^Key Laboratory of Bio-resources and Eco-environment of Ministry of Education, College of Life Sciences, Sichuan University, Chengdu, PR China.

^2^Department of Periodontics, West China Hospital of Stomatology, Sichuan University, Chengdu, PR China

6 Supplementary figures

**Supplementary Methods**

**Bacterial viability assays**

To monitor the growth of *E. coli* Rosetta (DE3) cells transformed with pET-28a or pET-28a-*BnTR1*, overnight cell cultures were diluted 1,000-fold and used to inoculate fresh LB broth containing 50 μg ml^-1^ kanamycin at 37°C. IPTG was added to mid-logarithmic phase cultures (OD_600_ = 0.5~0.6) at a final concentration of 0.1 mM. After induction for 1 hour, cultures were transferred and incubated at 37°C and 42°C. The same procedure was applied to *E. coli* C600 cells transformed with pBAD24, pBAD24-*BnTR1* or *BnTR1* mutants, except that the LB broth contained 100 μg ml^-1^ ampicillin with or without 0.1% L-arabinose and the cells were cultured at 30°C. Growth curves were generated by monitoring the optical density of cultures at 600 nm every 30 or 60 min.

To measure thermotolerance of *E. coli* Rosetta cells, mid-logarithmic phase (OD_600nm_=0.6) bacterial cultures were induced with 0.1 mM IPTG at 37°C for 1 hour. After induction, the cultures were diluted 10,000-fold and 1ml of each culture was transferred to 48.8°C for 0, 15, 30, 45, and 60 min. Each culture (100 μL) was then plated onto LB agar plates supplemented with 50 μg ml^-1^ kanamycin. The viable cells were counted after 12 hours incubation at 37°C.

**Bacterial two-hybrid assays**

Two-hybrid assays were performed using the BacterioMatchII two-hybrid system (Stratagene). Specifically, *BnTR1* was cloned into pBT as bait. *dnaK* and *rpoH* were inserted into the pTRG target plasmid as preys. The reporter cells were co-transformed with the corresponding plasmids and incubated at 37°C for 24 hours. Positive interactions were determined by growth on M9^+^ His-dropout broth with tetracycline/chloramphenicol and confirmed by growth in dual selective screening medium (5 mM 3-AT, 12.5 μg ml^-1^ streptomycin).

**Recombinant protein expression and purification**

For protein expression, overnight cultures were diluted 1:100 and grown to mid-exponential phase (OD_600nm_ = 0.6). The cells were induced with 0.2 mM IPTG overnight at 16°C. Culture pellets were harvested (7,000 rpm, 10 min) and re-suspended in 20 ml lysis buffer (50 mM NaH_2_PO_4_, pH 8.0, 300 mM NaCl, 10 mM Imidazole) with 1 mg ml^-1^ lysozyme. Suspensions were kept on ice for 30 min and supplemented with Protease Inhibitor (Roche) before sonication. Finally, samples were collected by centrifugation, and proteins were purified using a Protein Purification Kit (Qiagen). Briefly, nickel nitrilotriacetic acid superflow was used under native conditions for protein purification. Lysis buffers supplemented with 20 mM imidazole and 250 mM imidazole were used as washing and elution buffer, respectively. Protein purity was assessed using sodium dodecyl sulfate polyacrylamide gel electrophoresis (SDS-PAGE) and Coomassie staining after ultrafiltration using buffer (50 mM NaH_2_PO_4_, pH 8.0, and 300 mM NaCl) plus 5% [glycerol](app:ds:glycerol) and storage at -80°C. Purified DnaK, DnaJ and GrpE were commercially obtained (Enzo Life Sciences, Inc.).

***In vitro* assay of E3 ubiquitin ligase activity**

Purified BnTR1 or mutant forms (4 μg) from the supernatant of whole-cell extracts was incubated 2 μg with E1 (UBE1) (Boston Biochem), 4 μg E2 (UBCh5b) (Boston Biochem), and 2 μg His_6_-Ubiquitin (Boston Biochem) in the 20 μl reaction buffer (50 mM Tris-HCl pH 7.5, 2.5 mM MgCl_2_, 1 mM dithiothreitol, 1 mM ATP) at 30°C for 2h^1^. The reactions were terminated with SDS buffer plus β-mercaptoethanol and processed for 12% SDS-PAGE. Western blotting experiments were performed using the anti-His antibody to detect ubiquitin.

**Co-IP assays**

Purified BnTR1 and mutant forms (5 μg) was incubated with DnaK (1 μg) or His-σ^32^ (1 μg) in 50 mM Tris-HCl buffer, pH 7.5 supplemented with 0.15 M NaCl, 1 mM ATP and 1 mM DTT. The control group was set without DnaK or σ^32^ proteins in the reaction system. After incubation for 30 minutes at 30°C, the samples were incubated with 1 ug DnaK or σ^32^ antibody on ice for 1 hour. Samples used for immunoprecipitation with 20 μl protein A/G plus-agarose (Santa Cruz) was added. After incubation, the agarose beads were washed with 5 ml TBS buffer (50 mM Tris-HCl buffer, pH 7.5, and 0.15 M NaCl) and subjected to western blotting.

**RNA extraction and *q*RT-PCR**

For RNA extraction, *E. coli* Rosetta cells transformed with pET-28a, pET-28a-*BnTR1* (OD_600nm_ = 0.6), or pET-28a-*PUB18* (OD_600nm_ = 0.6) were induced with 0.1 mM IPTG at 37°C for 1 hour. Then the pET-28a and pET-28a-*BnTR1* cells were transferred and incubated at 37°C and 42°C for 1 hour, respectively. The pET-28a and pET-28a-*PUB18* cells were incubated at 37°C for 1 hour. Collected cells were lysed using Trizol (Invitrogen), and total RNA was isolated using phenol-chloroform and precipitated with ice cold isopropanol. Then the precipitations were further washed with 70% ethanol and dissolved in RNase-free water. Genomic DNA was removed using RNase-free DNase I. RNA samples were further purified using RNeasy Mini Kits (Qiagen) as previously study^2^. RNA concentration and quality were determined using A Nanodrop ND 1000 spectrophotometer (Thermo Fisher Scientific, Pittsburgh, PA, USA) and [agarose gel electrophoresis](https://www.baidu.com/link?url=SEoBrhtqJIgpHOaz-Swk2ewLL-hjo2d3zHQMPx3AzQ-sG4cYWJJ3HZxBcb_b-gJFheoLpD6HqeeDrwuLY13b1R8PR3Dsx_OA-NPU2EpAtCun71UfQ_CpDZaKBqbOkzyRI1UcGn3K6inFfUBuWYssF_&wd=&eqid=9d7166480000e1050000000555c04739). The isolated RNA was stored at −80°C before use.


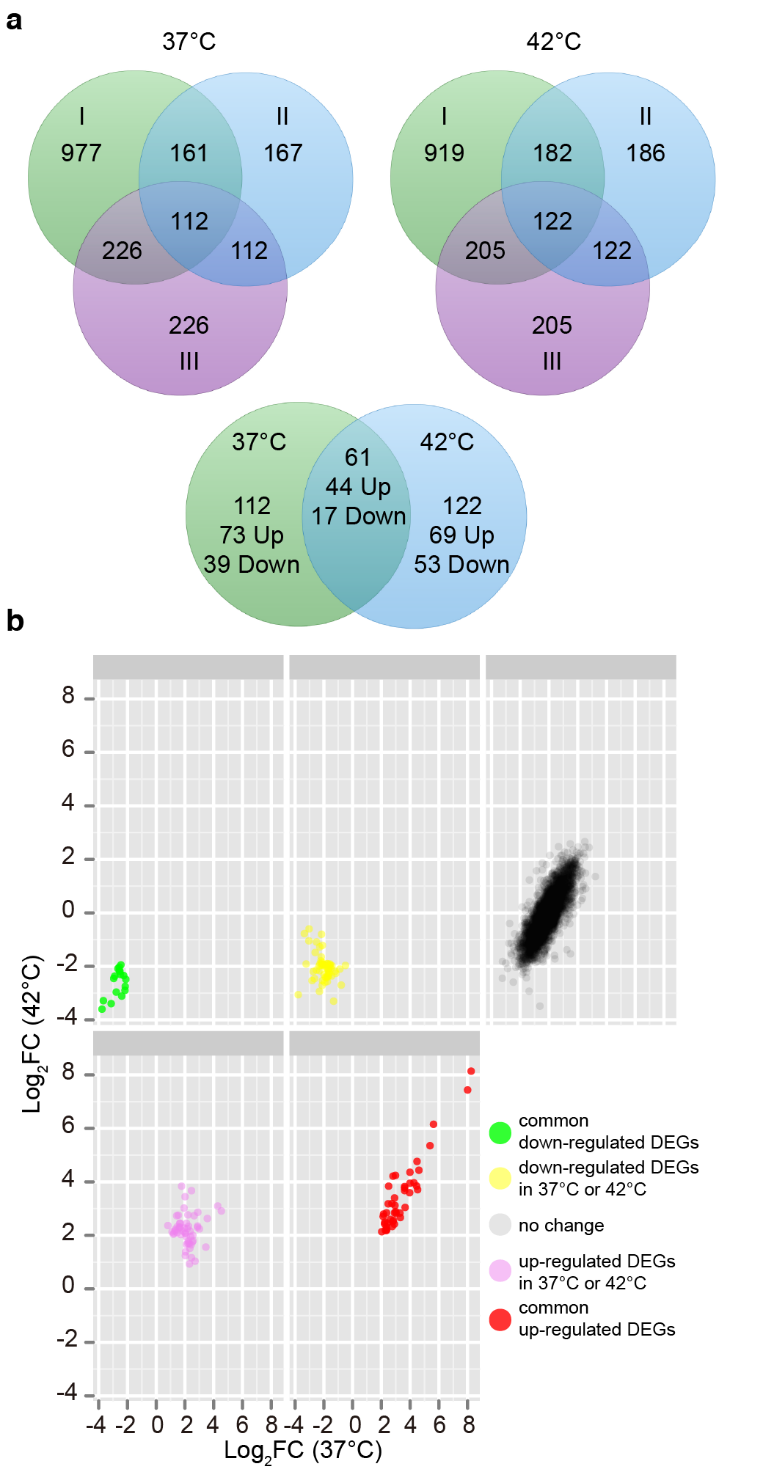


**Supplementary figure 1:** (**a**) The numbers of DEGs at 37°C and 42°C selected using different methods (top). The methods include I (Student’s *t*-test, Mann-Whitney *U*-test and fold-changes), II (Rank Product) and II (Limma). The common DEGs are shown at the bottom. (**b**) Distribution of DEGs at 37°C and 42°C.

**
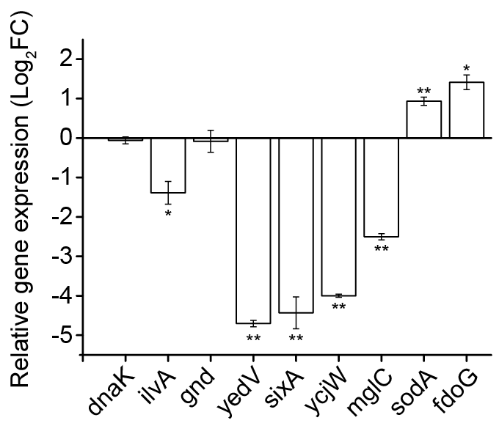
**

**Supplementary figure 2:** Comparison of transcription levels of selected genes from *E. coli* cells expressing pET-28a and pET-28a-*PUB18* at 37°C by *q*RT-PCR. The two-tailed Student’s *t*-test was applied for the comparison (**p*-value < 0.05 and ***p*-value < 0.001). The data are presented as means ± s.d. of three independent experiments.


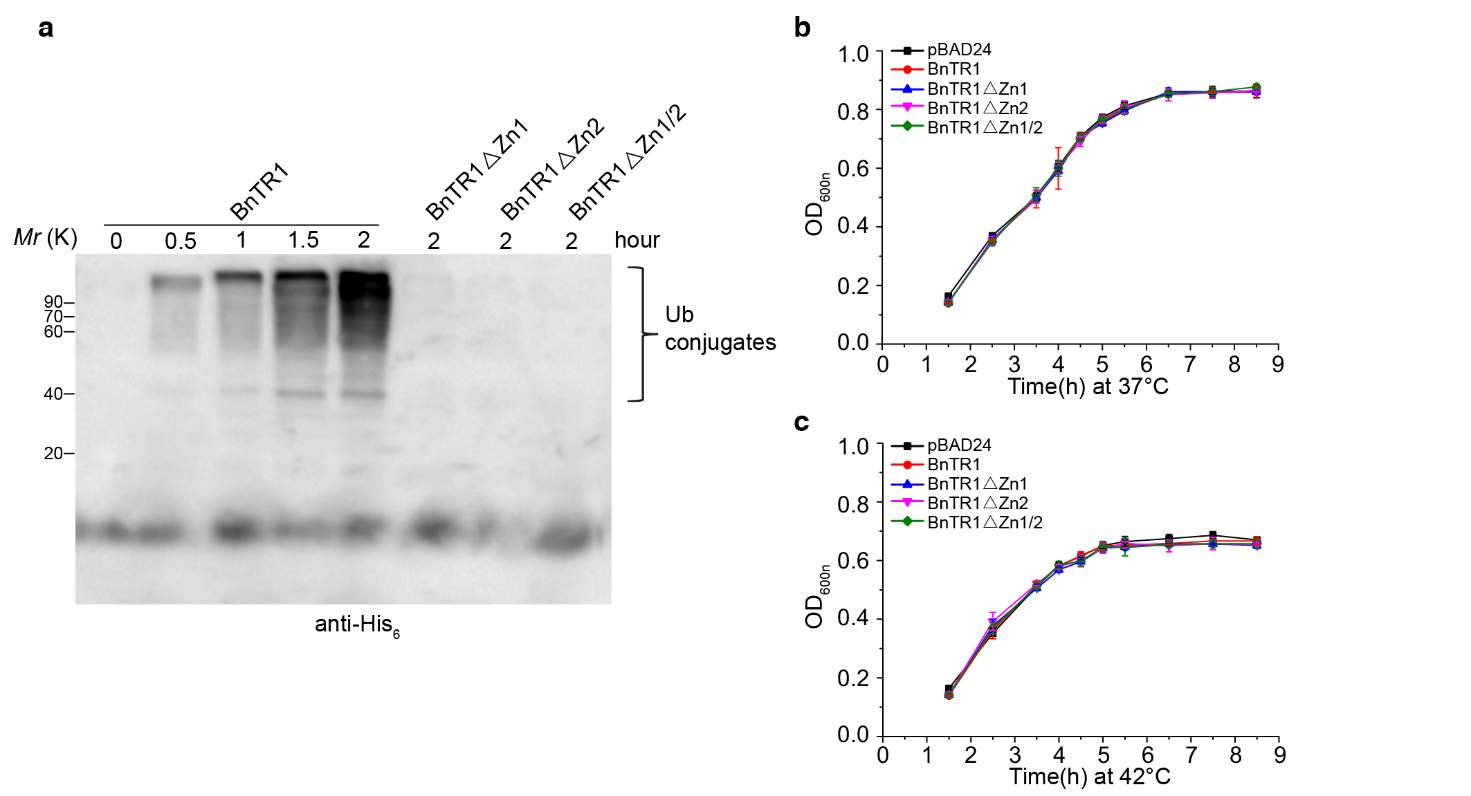


**Supplementary figure 3:** (**a**) *In vitro* E3 ubiquitin ligase activity of BnTR1 and mutant forms that extracted and purified from the supernatants of the cell lysate. Ubiquitin (Ub) conjugates bonds were detected by the anti-His_6_ for Ub. Growth curves of *E. coli* C600 cells harbouring BnTR1 mutations in the absence of L-arabinose at 37°C (**b**) and 42°C (**c**). The data are presented as means ± s.d. of three independent experiments. Full-length blots are presented in Supplementary Figure 6.


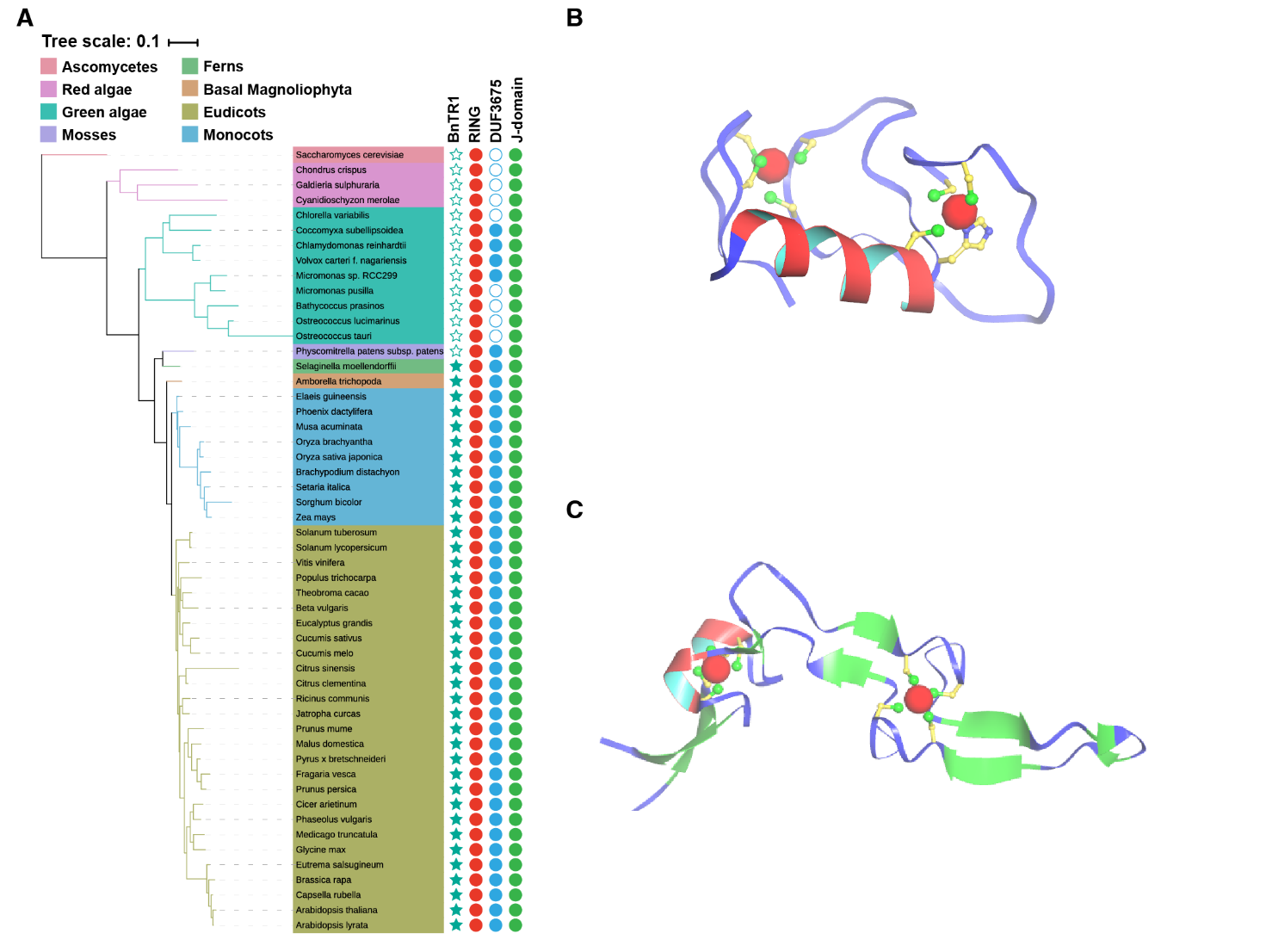


**Supplementary figure 4:** (**a**) A reconstructed phylogenetic tree of *Plantae* mapped with the presence (filled shapes) and absence (empty shapes) of full-length BnTR1, RING domain, DUF3675 domain and J-domain. (**b**) and (**c**) Ribbon diagrams of zinc fingers coloured with secondary structures in BnTR1 and DnaJ. The zinc ions are represented as balls coordinated with the corresponding amino acid residues. The zinc figure domain of BnTR1 was modelled using SWISS-MODEL^3^, and the DnaJ zinc finger was retrieved from Martinez-Yamout *et al.*^4^.


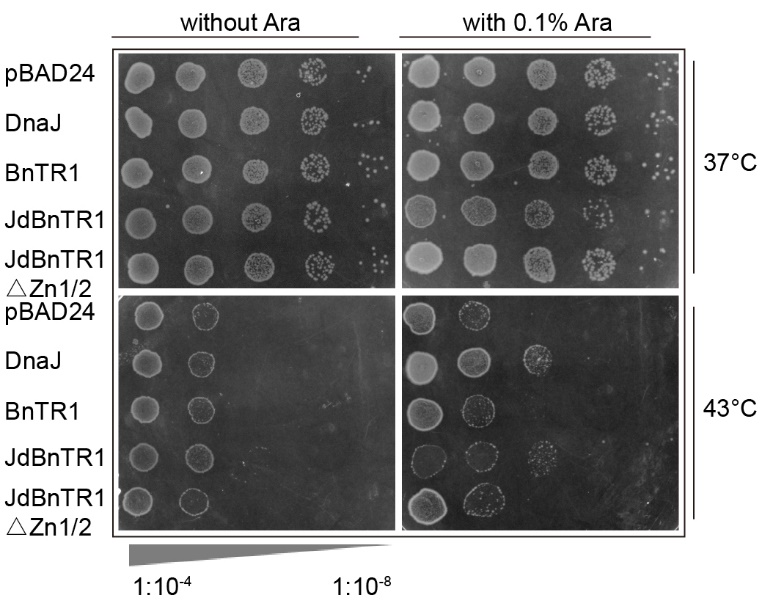


**Supplementary figure 5:** Complementation of temperature sensitive *E. coli* stains expressing pBAD24 DnaJ, BnTR1, JdBnTR1, and JdBnTR1 mutants in the presence of 0.1% L-arabinose inducer.

**Supplementary figure 6:** Full-length blots.


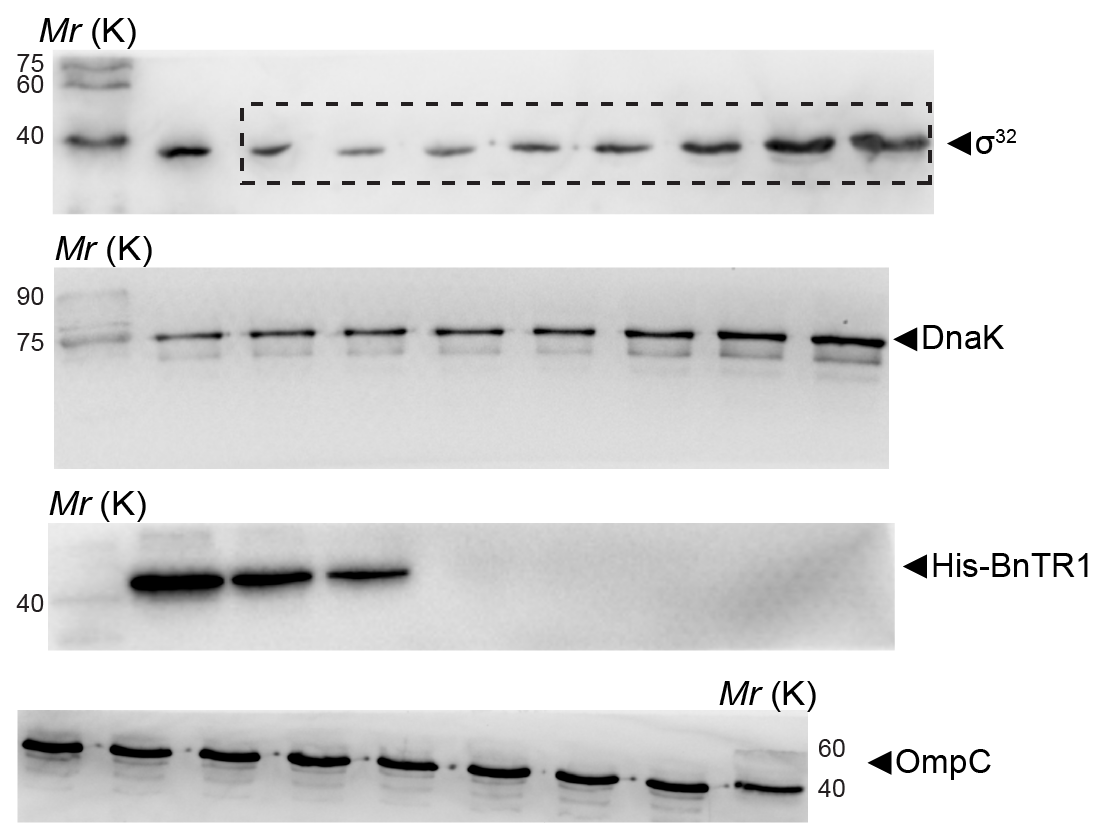
Full-length blots from Figure 2c

Full-length blots from
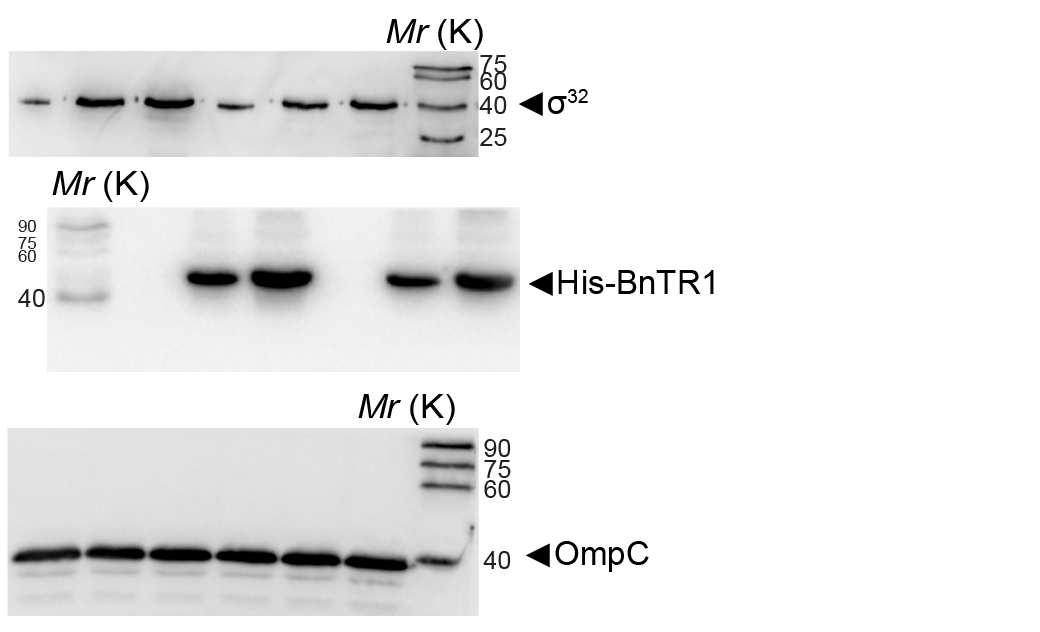
Figure 2d


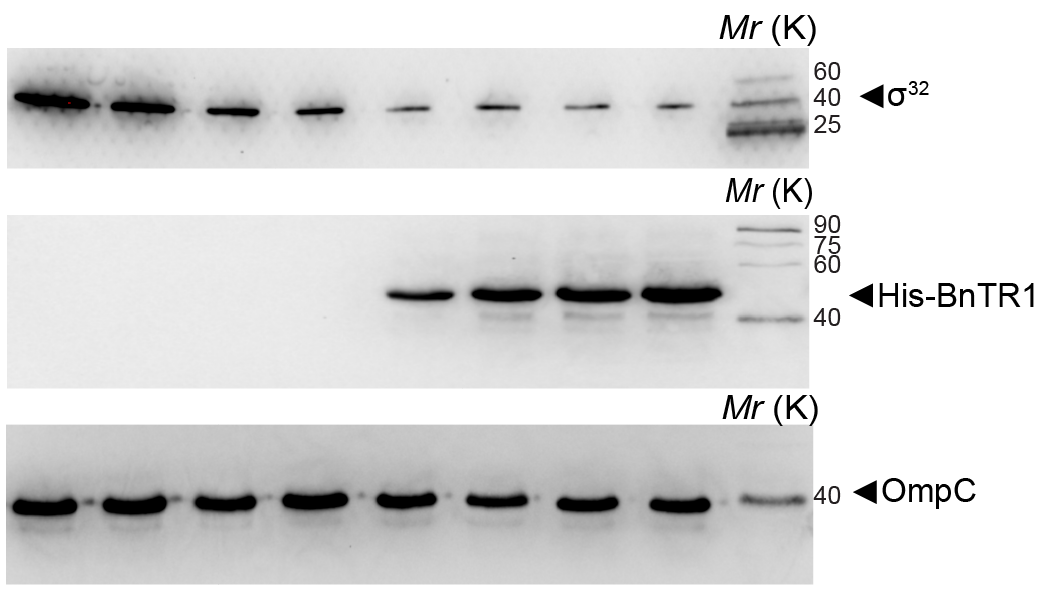
Full-length blots from Figure 2e


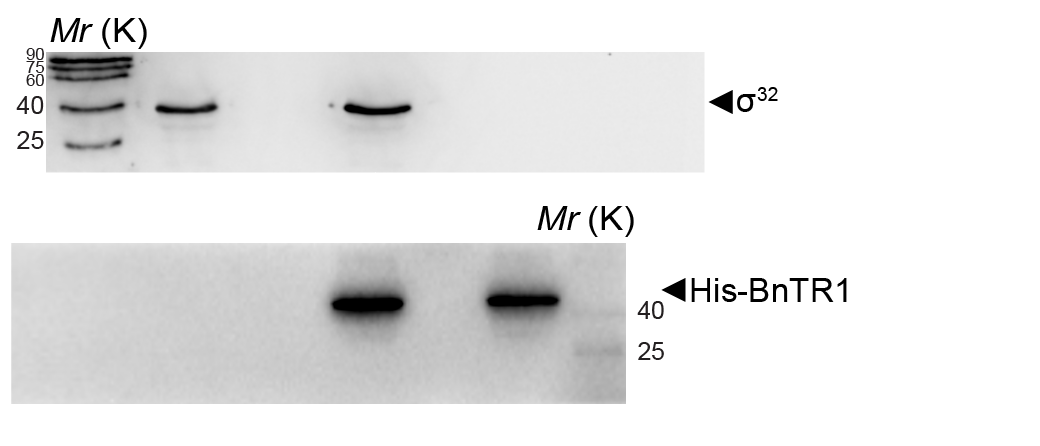
Full-length blots from Figure 2f


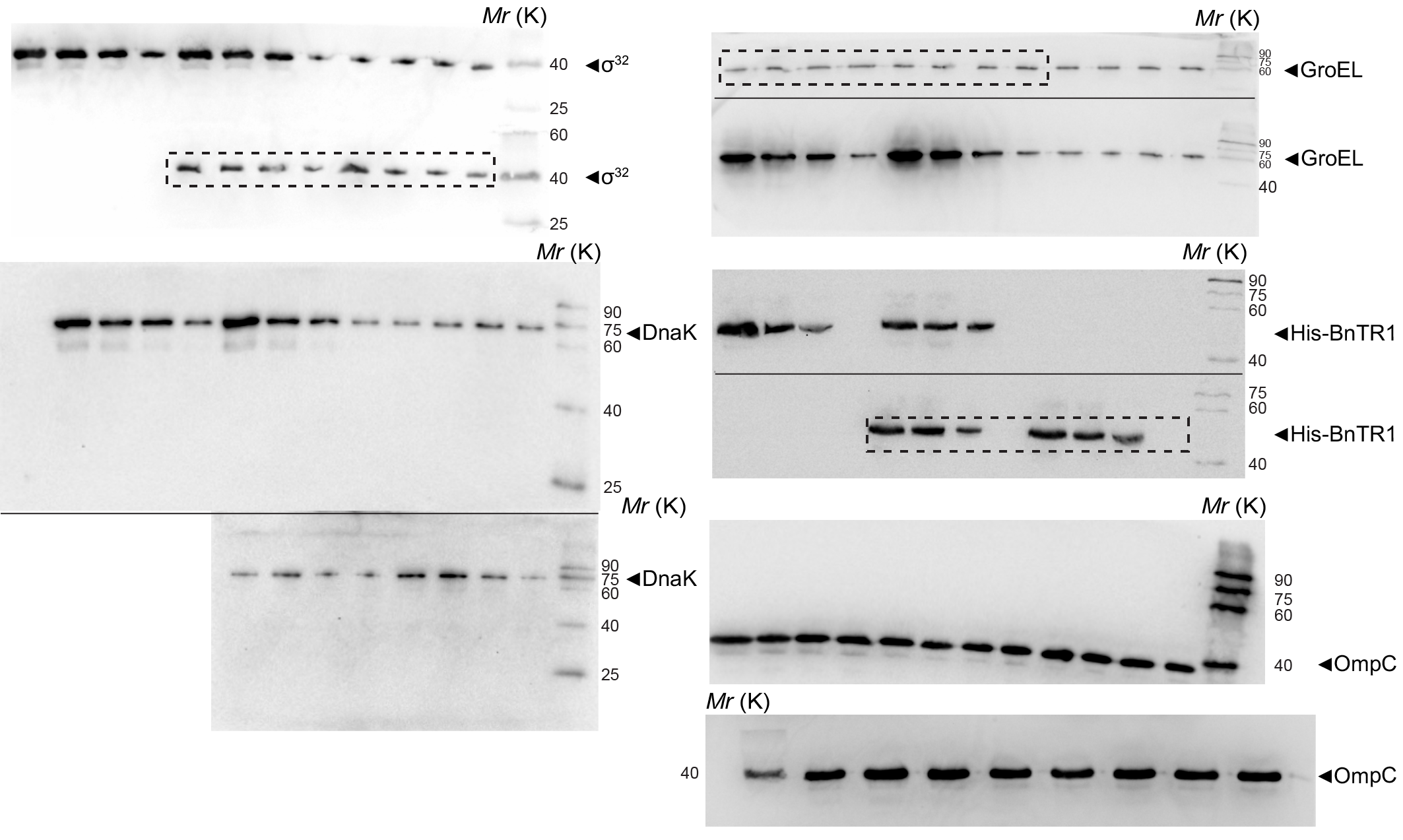
Full-length blots from Figure 3b


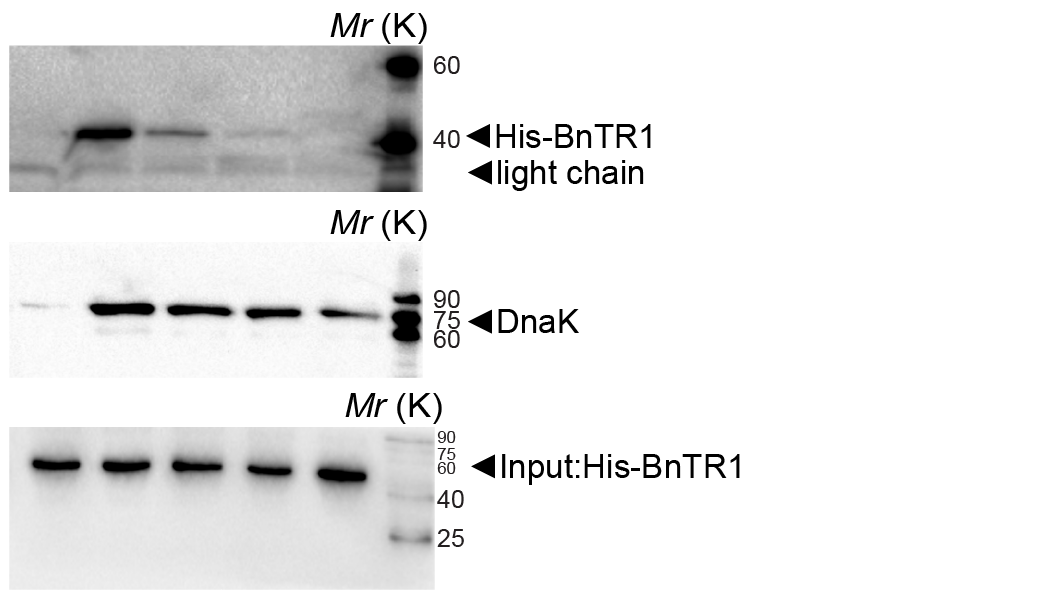
Full-length blots from Figure 4c

Full-length blots
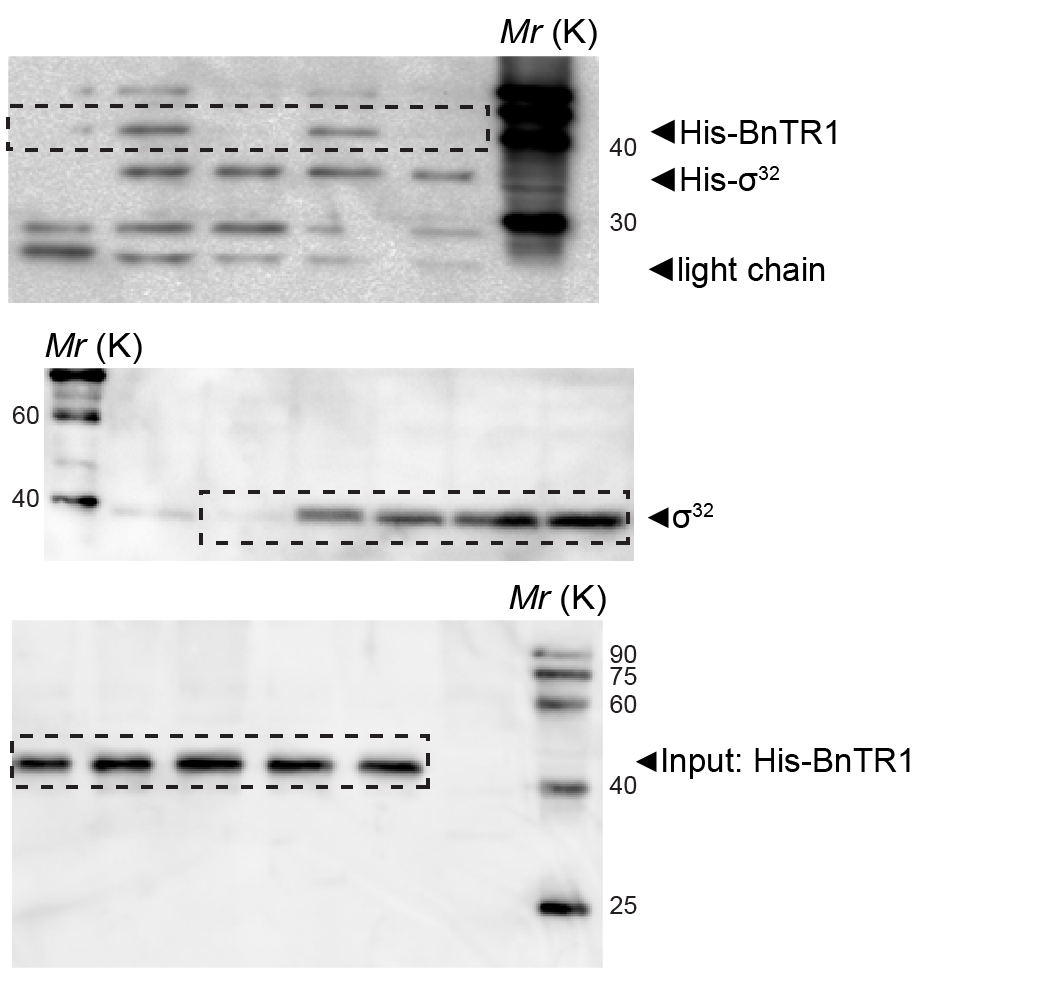
from Figure 4e


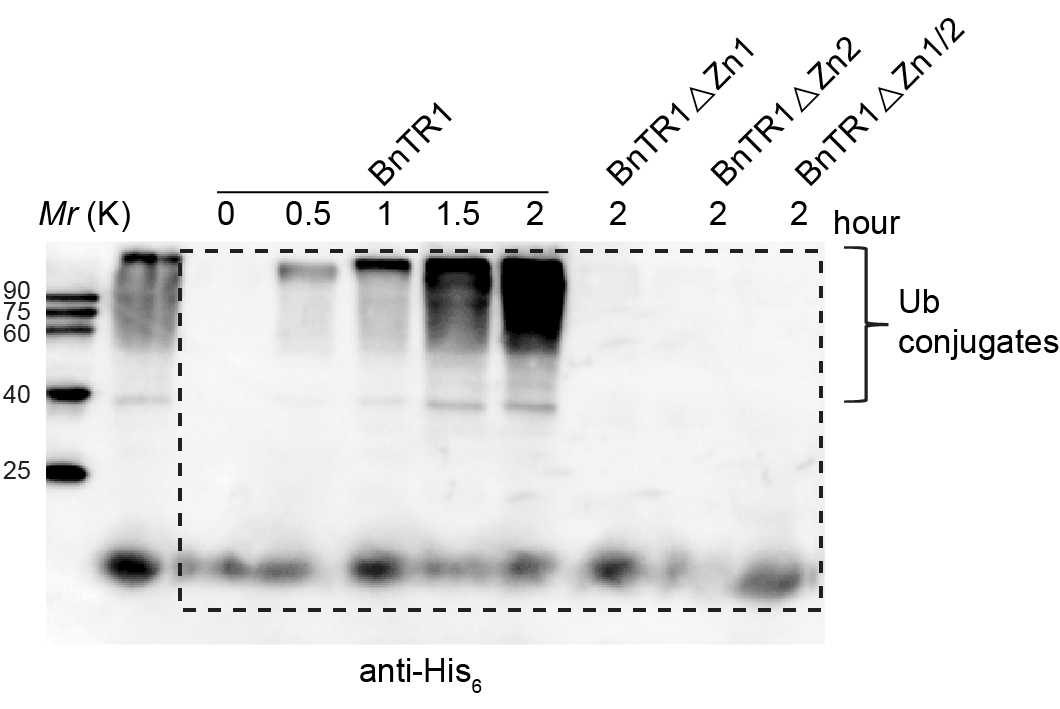
Full-length blots from Supplementary figure 3a

**References:**

1. Lu D, *et al.* Direct ubiquitination of pattern recognition receptor FLS2 attenuates plant innate immunity. *Science* **332**, 1439-1442 (2011).
2. Liu C, *et al.* Streptococcus mutans copes with heat stress by multiple transcriptional regulons modulating virulence and energy metabolism. *Sci Rep* **5**, 12929 (2015).
3. Martinez-Yamout M, Legge GB, Zhang O, Wright PE, Dyson HJ. Solution structure of the cysteine-rich domain of the Escherichia coli chaperone protein DnaJ. *J Mol Biol* **300**, 805-818 (2000).
4. Biasini M *et al*. SWISS-MODEL: modelling protein tertiary and quaternary structure using evolutionary information. *Nucleic Acids Res.* **42**(Web Server issue), W252-8 (2014).
